# Placental Cannabinoid Receptor Expression in Preterm Birth

**DOI:** 10.1155/2024/6620156

**Published:** 2024-05-07

**Authors:** Stepan Feduniw, Izabela Krupa, Katarzyna Łagowska, Piotr Laudański, Jacek Tabarkiewicz, Barbara Stawarz, Grzegorz Raba

**Affiliations:** ^1^Department of Gynecology, University Hospital Zurich, 8091 Zurich, Switzerland; ^2^Laboratory for Translational Research in Medicine, Centre for Innovative Research in Medical and Natural Sciences, Faculty of Medicine, University of Rzeszów, 35-310 Rzeszów, Poland; ^3^Chair and Department of Obstetrics, Gynecology and Gynecological Oncology, Medical University of Warsaw, Warsaw, Poland; ^4^Women's Health Research Institute, Calisia University, 62-800 Kalisz, Poland; ^5^OVIklinika Infertility Center, 01-377 Warsaw, Poland; ^6^Department of Human Immunology, Institute of Medical Sciences, Medical College of Rzeszów University, University of Rzeszów, 35-959 Rzeszów, Poland; ^7^Provincial Hospital in Przemysl, 37-700 Rzeszów, Poland; ^8^Medical College of Rzeszów University, University of Rzeszów, 35-315 Rzeszów, Poland

**Keywords:** cannabinoid receptor, CBR1, CBR2, ECS, preterm birth, PTB

## Abstract

**Background:** The cannabinoid receptor (CBR) plays a significant role in oogenesis, pregnancy, and childbirth. It might also play a significant role in preterm birth (PTB). The aim of the study was to investigate the association between the expression of the CBR in the placenta and the incidence of PTB.

**Methods:** This prospective, observational, multicentre preliminary study was conducted on placental samples obtained from 109 women. The study included 95 patients hospitalized due to the high risk of PTB. They were divided into two groups: Group 1, where the expression of the CBR1 and CBR1a was analyzed, and Group 2, in which we examined CBR2 expression. The control group, that is, Group 3, consisted of 14 women who delivered at term, and their placentas were tested for the presence of all three receptor types (CBR1, CBR1a, and CBR2).

**Results:** The study used reverse transcription and real-time PCR methods to assess the expression of CBRs in the placental tissues. The expression of the CBR2, CBR1, and CBR1a receptors was significantly lower in the placentas of women after PTB compared to those after term births, *p* = 0.038, 0.033, and 0.034, respectively.

**Conclusions:** The presence of CBR mRNA in the human placental tissue was confirmed. The decreased expression of CBRs could serve as an indicator in predicting PTB.

## 1. Introduction

Preterm birth (PTB) continues to pose a challenge in neonatal medicine, and it is the major cause of perinatal morbidity and mortality. Despite modern advances in obstetric and perinatal care, neonatal outcomes are still unsatisfactory due to high PTB rates [1]. PTB affects approximately 5–9% of births in Europe, 12–14% in the United States, and around 18% in Africa and Asia [2, 3]. PTB leads to severe neonatal complications, negatively affects the mental well-being of parents, and is associated with substantial economic costs due to the need for specialized medical support. According to the Eunice Kennedy Shriver National Institute of Child Health and Human Development Neonatal Research Network report on very low birth weight (less than 1500 g), the complications of PTB include respiratory distress (93% of infants), retinopathy of prematurity (59%), patent ductus arteriosus (46%), bronchopulmonary dysplasia (42%), late-onset sepsis (36%), necrotizing enterocolitis (11%), severe intraventricular hemorrhage (7–9%), and periventricular leukomalacia (3%) [4].

The etiology of PTB is complex and multifactorial. Key mechanisms leading to PTB include the early activation of the fetal hypothalamic–pituitary–adrenal axis in response to maternal and/or fetal stress, premature placental abruption, uterine expansion problems, cervical insufficiency, and inflammation, especially in the urogenital tract [5]. Preterm prelabor rupture of the membranes (PPROM) also contributes to a high incidence of PTB. PPROM is mainly triggered by inflammation associated with an infection, where inflammatory cytokines weaken the fetal membranes [6–9].

Inflammation is a crucial factor in both PTB and term birth [10]. Although a higher concentration of inflammatory cytokines was found in the amniotic fluid following delivery [11], Raba and Tabarkiewicz observed that inflammatory cytokines played a crucial role in over 80% of PTB cases, particularly in those occurring before 30 weeks of gestation [12]. Other causes of PBT include premature rupture of the membranes caused by an infection of the amniotic sac or alterations in vaginal bacterial flora [13, 14].

The endocannabinoid signaling system (ECS) includes the cannabinoid receptor (CBR) type 1 (CBR1) and type 2 (CBR2), along with their endogenous ligands (arachidonoylethanolamide and 2-arachidonoyl glycerol) for the CBRs. THC (*Δ*9-tetrahydrocannabinol) is one of the best-known exogenous activators of the CBR1 and CBR2. It is found in *Cannabis sativa* (marijuana), widely recognised as a psychoactive agent. Signaling pathways mediated by the CBR may affect cell proliferation, differentiation, and apoptosis in animal and human cells [15–17].

The CBR1 and its alternative splice variant form—the CBR type 1a (CBR1a), encoded by the *CNR1* gene—are G-protein-coupled receptors, predominantly found in the central nervous system, the heart, liver, uterus, testes, and the small intestine [18]. The CBR2, encoded by *CNR2*, shows a 44% amino acid sequence similarity to the CBR1 and is expressed in T and B lymphocytes, macrophages, hematopoietic cells, the brain, and other peripheral tissues, where it modulates the immune response [19–21].

CBRs are involved in both the male and female reproductive systems (the oviduct, uterus, and embryo) [22, 23]. The ECS plays a significant role in the regulation of oogenesis, embryo development, embryo transport, implantation, and placental development, as well as pregnancy and childbirth [24, 25]. ECS dysregulation might contribute to delayed embryonic development, poor blastocyst implantation, inhibited decidualization, compromised placentation, preeclampsia, ectopic pregnancy, and miscarriage [22, 24, 26]. While many studies showed that the ECS affected pregnancy outcomes, the precise role of the CBR is yet to be fully understood.

It is essential to look for new molecules and pathways playing a crucial role in the pathomechanism of PTB. In order to identify new markers of PTB, we should consider the potential value of the cannabinoid system. No human studies have been conducted on this subject, but evidence from animal models is promising. This present study was designed to investigate the link between placental CBR expression and PTB.

## 2. Materials and Methods

In this multicenter study, we analyzed placental samples collected in the Obstetrics Department at the Provincial Hospital of Przemyśl, the Department of Perinatology of the Medical University of Bialystok, and the Obstetrics Clinic of the Medical University of Lublin. The placentas were collected prospectively from women who gave birth between March 2004 and December 2012. The participants were divided into three groups. Group 1 consisted of women who experienced PTB, and their placentas were assessed for the expression of the CBR1 and CBR1a; Group 2 included women who also delivered preterm and whose placentas were examined for CBR2 expression. Some participants were included both in Groups 1 and 2. Group 3 was the control group; it comprised women who delivered at term, and their placentas were tested for the presence of all three receptors (CBR1, CBR1a, and CBR2).

### 2.1. Inclusion and Exclusion Criteria

The following inclusion criteria were applied to Groups 1 and 2: spontaneous birth ending in vaginal birth or emergency Cesarean section (C-section) between 22 weeks and 0 day and 36 weeks and 6 days of gestation. Group 3 included women who underwent spontaneous labour between 37 weeks and 0 day and 40 weeks and 6 days of gestation. All women included in the study were in active labour before delivery, exhibiting regular uterine contractions, at least two contractions/10 minutes resulting in cervical dilation or cervical effacement. All C-sections were performed based on emergency obstetric indications, and elective C-sections were excluded from the study. Other exclusion criteria encompassed complications diagnosed during the current pregnancy, such as multiple gestations, anemia, intrahepatic cholestasis of pregnancy, acute fatty liver of pregnancy, chronic kidney disease, autoimmune disorders, immunodeficiency, placenta previa, fetal growth restriction, HELLP syndrome, oligohydramnios, polyhydramnios, and other rare complications not otherwise specified. Pregnancies with confirmed fetal chromosomal or anatomical abnormalities, pregnancies conceived using in vitro fertilization, cases of intrauterine fetal demise, and iatrogenic terminated pregnancies were also excluded from the study. Moreover, samples with insufficient RNA quality were excluded from further analysis.

This study was conducted in accordance with the STROBE Statement–Checklist for cohort studies (Table S1) [27].

Informed consent was obtained from all participants, with the study design and objectives clearly communicated prior to enrollment. The study received the approval of the Bioethics Committee of the University of Rzeszów (reference number 05/10/2020). This study was conducted in accordance with the Declaration of Helsinki.

### 2.2. Placental Samples

Placental tissue samples were collected directly after the third stage of labour. The samples were obtained from four macroscopically unaffected parts of the placenta, one from each of the four quadrants of the decidua basalis—the maternal surface of the placenta. The samples were placed in a tube and stored at −86°C to maintain integrity until analysis. RNA isolation, then reverse transcription, and finally, real-time PCR were conducted to determine the expression of the mRNA of the target genes.

### 2.3. RNA Isolation

Total RNA was isolated from the placental tissue using the Gene MATRIX Universal RNA Purification Kit (EurX, Gdańsk, Poland) and supplemented with TRI-reagent (phenol equilibrated, stabilized: chloroform: isoamyl alcohol 25 : 24 : 1) (AppliChem GmbH, Darmstadt, Germany) and *β*-mercaptoethanol (Acros Organics, New Jersey, USA). The procedure was conducted according to the manufacturer's protocol. Three milligrams of each sample was homogenized using gentleMACS Tubes (Miltenyi Biotec GmbH, Bergisch Gladbach, Germany) and gentleMACS Dissociator (Miltenyi Biotec GmbH, Bergisch Gladbach, Germany). Total RNA quantity and concentration were measured with Nanodrop 2000 spectrophotometer (Thermo Fisher Scientific, MA, USA) and stored at −86°C. The RNA quantity included in the study ranged between 1.9 and 2.0, and the median RNA concentration for samples was 1000 ng/*μ*l.

### 2.4. Reverse Transcription

RNA extraction was followed by gDNA elimination, and reverse transcription was performed using the QuantiTect Reverse Transcription Kit (Qiagen, Hilden, Germany). One microgram of RNA was transcribed, with incubation during the reverse transcription procedure conducted in Labcycler 48 (SensoQuest GmbH, Göttingen, Germany). The obtained cDNA was then stored at −86°C.

### 2.5. Real-Time PCR

Real-time PCR procedure was performed in a thermal cycler Roche LightCycler® 480 Real-Time PCR System (Roche Diagnostics Ltd, Basel, Switzerland) with the use of Power-Up SYBR Green Master Mix (Applied Biosystems, CA, USA). The target genes in our study were *CNR1*, *CNR1a*, and *CNR2* encoding CBR1, CBR1a, and CBR2, respectively. *ACTB* (*β*-actin) and *GAPDH* (glyceraldehyde 3-phosphate dehydrogenase) served as reference genes. In a single reaction, 1 *μ*l of cDNA was used. Gene-specific primers projected in Primer-BLAST were used at a 10-*μ*M concentration (primer sequences are presented in Table 1).

The real-time PCR procedure was followed by standard curve preparation. The real-time PCR consisted of uracil-DNA glycosylase activation at 50°C for 2 min, initial denaturation at 95°C for 2 min, followed by 45 cycles of denaturation at 95°C for 15 s, and annealing at 60°C for 1 min. The last step was the melting curve performed to ensure reaction specificity. Specific expression levels were calculated using the 1/ΔCt algorithm, where ΔCt was the Ct value of the target splicing variant minus the mean of the Ct value of reference genes (Algorithm 1).

### 2.6. Statistical Analysis

Statistical analyses were performed using STATISTICA software, version 13.3.

Demographic data are presented as the mean (M) ± standard deviation (SD) and the percentage in each group. The Shapiro-Wilk test was applied to assess the normality of data distribution. For data following a normal distribution, the *t*-student test was used to compare the expression of receptors between the groups. For variables without the normal distribution, the Mann–Whitney *U* test was performed. The significance of qualitative variables was verified using the chi-square test. Furthermore, Spearman's rank correlation was performed to check the relationships between two quantitative variables. In all calculations, the level of significance was set at *p* value ≤ 0.05. The statistical power of the performed test for showing a correlation between CBR expression PTB was 1–ß = 0.9 (*ɑ* = 0.05).

## 3. Results

**Figure 1:**
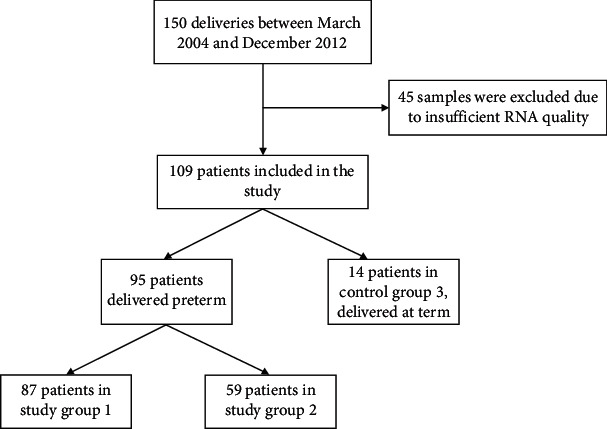
Flow chart of the study.

A total of 150 women were enrolled in the study, and 45 patients were subsequently excluded due to insufficient RNA quality in the placental samples. Ultimately, the study included 95 patients who experienced PTB, divided into two groups: 87 in Group 1 for the analysis of CBR1 and CBR1a expression and 59 in Group 2 for the analysis of the CBR2. Notably, some of the patients were included in both groups. The control group consisted of 14 women who delivered at term, and their placentas were tested for the presence of all three receptors (CBR1, CBR1a, and CBR2). The participant selection process is shown as a flow chart in Figure 1.

Population characteristics were separately analyzed, using two independent analyses between Group 1 and Group 3 and between Group 2 and Group 3. The findings are summarized in Table 2.

**Figure 2:**
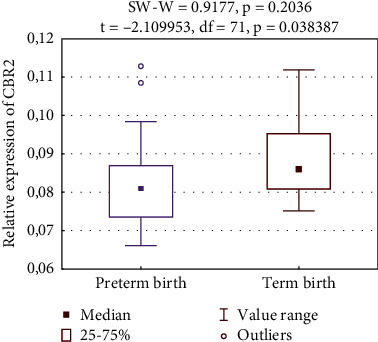
Relative expression of the CBR2.

Our findings revealed that the relative average expression of the CBR2 was significantly lower in Group 2 than in Group 3, 0.0816 ± 0.0098 vs. 0.0878 ± 0.01, respectively (*p* = 0.038). Differences in relative CBR2 expressions are presented in Figure 2.

In our analysis, the expression levels of CBRs were compared between 59 patients who delivered preterm from Group 2 (one) vs. 14 patients from the control group, Group 3 (zero). Statistical results, including the *p* value, *t*-statistics and degrees of freedom are shown in Figure 2.

A strong correlation was found between the expression of the CBR1 and CBR1a (R Spearman = 0.969, *t*(*N*‐2) = 39.14, *p* < 0.001). Since CBR1a mRNA is the alternative splice variant form of the CBR1, we focused exclusively on the results of CBR1 expression in the present analysis.

**Figure 3:**
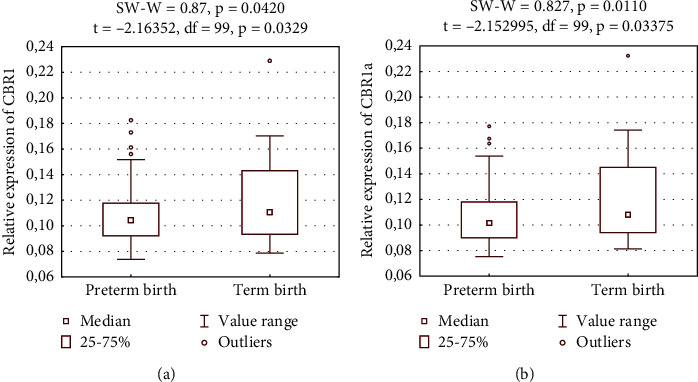
Relative expressions of (a) the CBR1 and the (b) CBR1a.

The expression of the CBR1 was found to be significantly lower in Group 1 than in Group 3, that is, 0.108 ± 0.022 versus 0.124 ± 0.041, respectively (*p* = 0.033). Similarly, CBR1a expression was also lower in Group 1 versus Group 3 (0.106 ± 0.022 vs. 0.122 ± 0.043, respectively (*p* = 0.034)). Differences in relative CBR1 and CBR1a expressions are presented in Figures 3(a) and 3(b).

We compared 87 patients who delivered preterm from Group 1 (one) and 14 control patients from Group 3 (zero). Statistical results are presented as the *p* value, *t*, and df of performed tests in the diagram. We performed an additional analysis of PTB risk factors, including the previous history of PTB, previous history of miscarriages, parity, urinary tract infections, vaginitis, other infections, leucocythemia, fetal growth restrictions, and poor socioeconomic status. Nonetheless, multivariate analysis showed no significant results.

**Figure 4:**
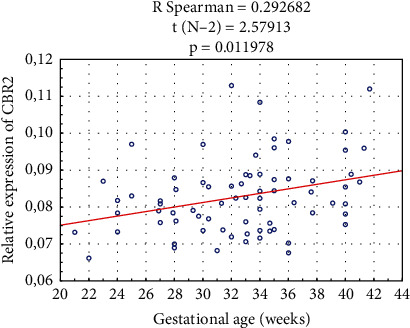
Correlation between the expression of the CBR2 and gestational age at delivery.

We observed a positive correlation between the relative expression of the CBR2 and pregnancy duration (R Spearman 0.29, *p* = 0.012) (Figure 4). No other correlation of steroid use, tocolysis, smoking, alcohol use, infection, socioeconomic status, or parity was observed.

Logistic regression was performed to establish the risk factors of PTB in the study population. Differences were noted only between the expression of CBRs and occurrence of PTB. The odds ratios (OR) for the CBR1, CBR1a, and CBR2 were 0.01 (95% CI: 0.01–0.69), 0.01 (95% CI: 0.01–0.80), and 0.01 (95% CI: 0.01–0.52), respectively. The results of the regression are presented in Table 3.

## 4. Discussion

In this study, we established a positive correlation between the expression of the CBR in the analyzed postpartum human placental tissues and PTB. First of all, we confirmed that CBRs were present in the placental tissue after delivery. Moreover, we demonstrated a significant decrease in the expressions of the CBR1, CBR1a, and CBR2 in the placentas obtained from women after preterm deliveries, compared to placentas from women after term deliveries, suggesting a potential role of the receptors in the mechanisms leading to PTB. Last but not least, a negative correlation occurred between pregnancy duration and the CBR2, perhaps due to the two main effects of CBRs, that is, their impact on muscle tissue and their role in inflammatory processes.

Previous research by Raba and Tabarkiewicz showed a correlation between the level of cytokines influencing PTB [12], and multiple authors described the effect of CBR2 stimulation on the release of cytokines. Moreover, the CBR may be stimulated by several cytokines (IL-1, IL-4, IL-10, IL-6, TNF-a, IL-8, MIP-1 (CCL3 and CCL4), and RANTES (CCL5)) [28–30]. This interplay suggests a complex relationship between cytokine activity and CBR function, and further studies are needed to establish the correlation between specific cytokines and CBR2 stimulation in pregnant women. An algorithm based on the measurement of the concentration of specific cytokines might help predict PTB [12]. The ECS modulates the action of leukocytes by stimulating the CBR2 presented on the leukocyte cells [31], which then inhibit the inflammatory response [32, 33]. This mechanism was observed in utero in patients with adenomyosis [34, 35]. PTB might be associated with inflammation caused by an intraamniotic infection [36, 37], but inflammation also plays a role in physiological term birth when no infection is present [33, 38]. A decrease in the placental expression of the CBR2 might initiate inflammation, leading to a complete loss of the receptors [39].

Some researchers conducted studies on CBR expression in the human model analyzing its role in the inflammatory processes in patients with endometriosis [34, 35, 40, 41]. Bouaziz et al. established that cannabinoids might be a highly effective treatment for women with endometriosis because of their complex action: the effect on the central and peripheral nervous system, suppression of neuropathic and inflammatory pain, psychological impact, the levels of hormones affecting the perception of pain, and the effect on the expression of CBRs, enzymes, and ligands [42].

Previous studies on animal models revealed similar results to those of our study, reinforcing the potential relevance of CBRs in PTB. Sun et al. observed that a lower expression of the *CNR2* gene correlated with PTB in mice [18]. Another study on the mouse model also showed that decreased CBR2 and increased CBR1 expression were associated with PTB. In that study, PTB occurred due to preterm induction with a lipopolysaccharide [33]. Wang, Xie, and Dey showed a positive correlation between decreased CBR1 expression in the placenta of mice and PTB [43].

Only a few studies have been published in recent years where the correlation between labour and CBR expression was observed. Park et al. found a high expression of the CBR1 in the human placenta and an even higher expression in the amniotic epithelium, reticular cells, and the cells of the maternal decidua layer [44]. Kozakiewicz et al. showed that the ECS played a role in childbirth via the expression of the CBR1 [45]. However, the role of CBR expression as a potential predictor for PTB in humans remains unexplored. This information might have important implications for clinical practice. Therefore, further studies are needed to assess the expression of CBRs during pregnancy.

Furthermore, based on a study conducted in humans, it seems that a decrease in CBR1 and CBR1a mRNA levels might be connected with placental disorders, such as preterm placental abruption, leading to preterm deliveries or the intrauterine death of the fetus [46]. However, our study did not show such effects, as no changes in intrapartum CBR mRNA levels were observed, indicating a complex interaction that warrants further investigation.

The expression of CBRs might be affected by the use of tocolytic agents or corticosteroid therapies, commonly used for fetal lung maturation. Research by Pagano et al. highlighted the possible tocolytic effects of cannabis consumption [47]. There are no studies directly linking tocolysis administration with changes in CBR expression. Similarly, the impact of betamethasone and dexamethasone on CBR expression in the placenta has not been explored. Interestingly, a study on broilers indicated an elevated expression of the CBR1 in the broiler hypothalamus after 72 h of administration [48]. However, the effects on the hypothalamus and placenta might differ, and further studies are needed to evaluate the influence of those factors. Our study did not reveal any significant influence of tocolytic or steroid administration during pregnancy.

In routine medical practice, fetal fibronectin (fFN) and phosphorylated insulin-like growth factor-binding protein-1 (phIGFBP-1) are used in the negative prediction of PTB [49, 50]. Nevertheless, the positive predictive value of these methods is very low. Moreover, there are ongoing studies on several other biomarkers which might help predict PTB [38, 51, 52]. First of all, inflammatory markers are analyzed, such as the proinflammatory interleukins of cytokines found in the maternal serum [51] or in cervical secretions [53]. The study by Laudanski et al. showed that the levels of IGFBP-1, Eotaxin-1, BLC, BDNF, and MIP-1d measured in the serum might serve as predictive indicators for preterm labour. These biomarkers might distinguish between actual and false cases of PTB [51]. In another study, Laudanski et al. demonstrated a predictive value of MIP-3b/CCL19 serum levels [52]. The correlation of currently known biomarkers and CBR expression might help improve PTB prediction and, subsequently, neonatal outcomes. Using artificial intelligence methods, such as machine learning, might help refine the prognostic value of the existing clinical risk factors of PTB, especially in combination with biomarker analysis [54, 55]. Moreover, Villar et al. proposed the phenotypic classification of PTB [56], where identifying and classifying patients according to their distinct phenotypes could improve the management of PTB.

Identifying decreased perinatal CBR expression in preterm delivery placentas might help doctors improve PTB prediction and, thus, improve neonatal outcomes. However, chorionic villus sampling and other placental biopsies are invasive procedures, associated with 3–4% pregnancy loss and an increased incidence of PTB, according to the International Society of Ultrasound in Obstetrics and Gynecology (ISUOG) [57]. Moreover, the appropriate timing for placental tissue sampling is unknown and difficult to estimate because more than one placental biopsy could be related to a noticeably higher risk of miscarriage or PTB. In order to better assess the placental expression of CBR, a noninvasive test could be more practical. Liquid-based cytology could be used as a noninvasive perinatal method of measuring the expression level of CBR in decidual cells during pregnancy [58, 59]. Several analyses of the cervicovaginal fluid showed the potential predictive value of this examination in predicting PTB [60, 61], suggesting that a technique like liquid-based cytology might provide clinicians with information on physiological changes throughout pregnancy. This approach might allow for an identification of a decrease in CBR expression, signaling the onset of PTB. However, further research is needed to validate this hypothesis.

While previous research showed that the expression of the CBR was related to labour, this is the first study confirming the involvement of the CBR in preterm labour in the human placentas.

The limitation of the study is the size of the study groups. The study sample size is large enough to draw conclusions. However, a larger population would strengthen our findings. Another limitation is that the gestational age at which the placental tissue was collected varied considerably. The aim of the study was to establish the difference between PTB placentas and placentas of term births. While we showed that there was a correlation between CBR expression and pregnancy duration, a more detailed temporal analysis of the difference in CBR expression, for example, at every week of gestation, would deepen our understanding of the role of the ECS in PTB.

The practical significance of this study is based on the confirmed link between the reduced expression of the CBR1, CBR1a, and CBR2 and the increased likelihood of PTB. These findings indicate that the receptors might serve as biomarkers for predicting PTB. CBRs could be included in prenatal screening programmes to identify pregnancies at an elevated risk of PTB and allow for the early adoption of preventative interventions. Furthermore, gaining a deeper understanding of the function of CBRs in PTB could potentially lead to the creation of innovative therapeutic strategies, potentially using CBR expression modulation to delay or prevent PTB. However, further research is needed to confirm the practical application of these findings.

Future research should aim to confirm the results of this preliminary study by involving a larger and more diverse cohort, reflective of the general population. This should encompass women from different geographic regions, ethnicities, and socioeconomic levels, with various medical backgrounds. Such comprehensive research would serve to validate the initial findings and adjust for any confounding factors. Longitudinal studies are essential for determining the consistency of changes in CBR expression over time and their value for PTB prediction. Exploring the influence of CBRs on PTB risk might reveal novel preventive and treatment strategies. Furthermore, investigating the interaction between CBRs and other well-known risk factors for PTB could result in the development of a comprehensive risk assessment model, hence improving the overall applicability and impact of our research findings on clinical practices and prenatal care strategies.

## 5. Conclusions

The present research revealed a significant decrease in the expression of the CBR1, CBR1a, and CBR2 in the placentas following PTB, in contrast to term births. These findings hold profound implications for clinical practice, indicating the potential for developing a new biomarker profile using CBR expression levels for PTB prediction. An innovative estimation technology has the potential to greatly transform prenatal care. It could help healthcare personnel detect high-risk pregnancies and take appropriate actions, ultimately improving neonatal outcomes.

The ethical considerations surrounding the measurement of CBR expression in the placental tissue throughout pregnancy necessitate careful examination. However, the insights gained provide a compelling rationale for further research. There is a pressing need for additional studies aimed at developing noninvasive or minimally invasive techniques for evaluating CBR expression that could be implemented in clinical settings.

The expression of CBRs in the placenta shows promise as a reliable marker of the risk of PTB. This highlights the significance of the ECS in pregnancy and childbirth. Our findings contribute to the growing body of research on prenatal biomarkers, introducing a possible new area of study that has the potential to enhance prenatal diagnosis and therapies. Therefore, further studies are needed to assess the expression of the CBR during pregnancy.

## Figures and Tables

**Algorithm 1:**
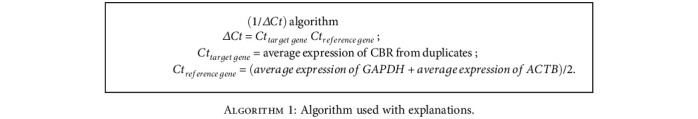
Algorithm used with explanations.

**Table 1:** Primer sequences.

**Target gene**	**Primer sequences (5**′**-3**′**)**
*ACTB*	F: 5′-CTGGAACGGTGAAGGTGACA-3′ R: 5′-GTAACAACGCATCTCATATTTGGAA-3′

*GAPDH*	F: 5′-AGAAGGCTGGGGCTCATTTG-3′ R: 5′-TGATGGCATGGACTGTGGTCAT-3′

*CNR1*	F: ′-TCATTAAGACGGTGTTTGCATTCT-3′ R: 5′-CGTGTCGCAGGTCCTTACTC-3′

*CNR1a*	F: 5′-TGCAGAGCTCTCCGTAGTCA-3′ R: 5′-TGGTCCTCGGGACAGAAG-3′

*CNR2*	F: 5′-TCATCTACACCTATGGGCATGTTCT-3′ R: 5′-CCTCATTCGGGCCATTCC-3′

**Table 2:** Characteristics of the study population.

**Factors**	**Group 1** **n** = 87**(%)** **M** ± **SD**	**Group 2** **n** = 59**(%)** **M** ± **SD**	**Group 3** **n** = 14**(%)** **M** ± **SD**	**p** **value** **Group 1 vs. Group 3**	**p** **value** **Group 2 vs. Group 3**
Age (years)	29.4 ± 6.4	29.7 ± 6.1	27.4 ± 4.8	0.31	0.22
Gestational age at delivery (hbd)	30.8 ± 3.9	30.9 ± 4.0	39.8 ± 1.3	< 0.001	< 0.001
Birth weight (g)	1706.2 ± 829	1749.8 ± 851	3470.0 ± 452	< 0.001	< 0.001
Parity					
1	52 (60)	37 (63)	12 (86)		
2	25 (29)	19 (32)	2 (14)	0.43	0.46
> 3	10 (11)	3 (5)	0		
History of PTB	14 (16)	8 (14)	1 (7)	0.38	0.49
History of miscarriages	9 (10)	6 (10)	1 (7)	0.71	0.72
History of stillbirth	5 (6)	2 (3)	0	0.12	0.46
Mode of delivery				0.46	0.59
Vaginal delivery	75 (86)	52 (88)	13 (93)		
C-section	12 (14)	7 (12)	1 (7)		
Height of the mother (cm)	163.7 ± 5.1	164.1 ± 5.2	165.4 ± 4.5	0.26	0.4
Weight before pregnancy (kg)	60.7 ± 10.0	62.7 ± 10.3	59.9 ± 10.1	0.8	0.43
Weight at delivery (kg)	68.9 ± 11.5	70.5 ± 12.2	73.9 ± 13	0.17	0.38
BMI before pregnancy (kg/m^2^)	21.7 ± 5.5	22.7 ± 5.3	21.8 ± 4.4	0.99	0.61
Temperature at delivery (C)	36.7 ± 0.4	36.7 ± 0.4	36.5 ± 0.4	0.14	0.064
Hemoglobin at delivery (mg/dl)	11.4 ± 1.1	11.4 ± 1.1	12.3 ± 1.2	0.02	0.035
White blood cell count at delivery (10^9^/L)	13.9 ± 5.8	12.4 ± 4.1	10.7 ± 3.2	0.092	0.23
Average FHR on admission (b/min)	144.3 ± 9.2	144.4 ± 10.1	144.3 ± 6.6	0.98	0.95
Maternal heart rate on admission (b/min)	79.8 ± 8.0	79.6 ± 6.0	82.9 ± 10.8	0.23	0.15
Use of tocolysis	40 (46)	27 (46)	2 (14)	0.018	0.022
Use of steroids	29 (33)	19 (32)	1 (7)	0.027	0.037
Antibiotics	28 (32)	19 (32)	3 (21)	0.41	0.42
Diabetes					
Pregestational diabetes mellitus	0	0	1 (7)	0.012	0.039
Gestational diabetes mellitus	3 (4)	2 (3)	1 (7)	0.51	0.53
Pregnancy hypertension	2 (2)	1 (2)	0	0.57	0.51
Smoking in pregnancy	14 (16)	12 (20)	2 (14)	0.86	0.61
Alcohol in pregnancy	7 (8)	5 (9)	3 (21)	0.12	0.16
Low socioeconomic status	9 (10)	5 (9)	0	0.093	0.14

**Table 3:** Logistic regression for preterm delivery.

	**OR (95% CI)**
Maternal age	1.06 (0.96–1.17)
Cesarean section delivery	1.06 (0.28–3.96)
Parity	3.39 (0.81–14.2)
History of preterm birth	2.31 (0.29–18.5)
White blood count on admission	1.18 (0.97–1.43)
CRP on admission	1.76 (0.85–3.66)
Gestational diabetes	0.46 (0.05–4.24)
CBR1	0.01 (0.01–0.69)
CBR1a	0.01 (0.01–0.8)
CBR2	0.01 (0.01–0.52)

## Data Availability

Research data are not shared.
